# Sputum host cytokine signatures for diagnosis of TB in children and adults

**DOI:** 10.3389/fimmu.2025.1652719

**Published:** 2025-10-14

**Authors:** Joseph Mendy, Edward Coker, January Weiner, Gian van der Spuy, Novel N. Chegou, Jayne S. Sutherland

**Affiliations:** ^1^ Vaccines and Immunity Theme, Medical Research Council Unit The Gambia at the London School of Hygiene and Tropical Medicine, Banjul, Gambia; ^2^ Core Unit for Bioinformatics, Berlin Institute of Health at Charité – Universitätsmedizin Berlin, Berlin, Germany; ^3^ South African Medical Research Council Centre for Tuberculosis Research, Division of Immunology, Department of Biomedical Sciences, Faculty of Medicine and Health Sciences, Stellenbosch University, Cape Town, South Africa

**Keywords:** tuberculosis, diagnostics, cytokines, sputum, host biomarkers

## Abstract

**Background:**

Tuberculosis (TB) still remains the world’s leading infectious disease killer. New screening and diagnostic tests are urgently needed. We have previously identified a 3-marker host protein signature with high accuracy for TB in sputum from adult Gambian patients. The aim of this project was to analyse host sputum markers in a larger cohort of adults and children with presumptive TB from The Gambia, and to determine the global applicability of such a signature in samples from South Africa, Vietnam and Peru.

**Methods:**

Sputum samples were collected at baseline from all symptomatic participants and used for routine diagnostics and biomarker evaluation. Samples were also collected at 1 and 2 months after anti-TB treatment initiation from those who were subsequently found to have TB. For biomarker evaluation, an aliquot of sputum was digested with an equal volume of Sputolysin^®^, incubated for 15 minutes at RT, centrifuged, and the supernatant analysed using multiplex cytokine arrays.

**Results:**

For Gambian participants (n=428 TB and n=313 other respiratory diseases (ORD)) an 8-marker signature was able to differentiate between smear and culture positive TB from ORD with an AUC of 0.77, while a 3-marker signature classified smear negative TB with an AUC of 0.74. Additionally, a 4-marker signature could discriminate between children (<18 years) with TB (n=17) and those with ORD (n=23) with an AUC of 0.87, sensitivity of 82% and specificity of 87%. A 5-marker global signature was identified with an AUC of 0.71.

**Conclusion:**

This study describes the use of host markers in sputum for development of a rapid triage test for pulmonary TB in adults and children. Whilst the results are promising for regional-specific and treatment response signatures, our pilot data do not support development of a global test for TB triage based on sputum host signatures.

## Introduction

Tuberculosis (TB) is the number one infectious disease killer worldwide, with over 1 million deaths in 2023 (1). There are an estimated 10 million new cases each year, but only around 7 million of these have a laboratory confirmation (1). The 2.7 million ‘missed’ cases are due to barriers like access to care and screening deficiencies that prevent detection and treatment.

Whilst there are several new TB diagnostic tests in the pipeline, current tests rely on detection of the pathogen such as sputum smear microscopy, GeneXpert Ultra and liquid culture. However, smear microscopy has low sensitivity (2), while culture (the gold-standard test) has high sensitivity but also high cost, infrastructure requirements and long turnaround time for results (2). GeneXpert Ultra generates results in 2 hours, with high sensitivity and specificity, but its use is also impeded by high cost and infrastructure requirements (2).

Due to the limitations associated with current conventional diagnostic methods, particularly for people living with HIV (PLWH) and children, WHO has prioritised developing a rapid screening test for TB in healthcare settings (1). Additionally, point-of-care (POC) tests that can readily determine response to treatment are urgently needed to replace sputum smear and culture due to the issues highlighted above. Promising TB triage tests include improvements on established technologies, such as chest radiography with computer-aided detection (CAD) analysis, which may enable screening without the need for a skilled reader (3). Host biomarkers also hold promise for translation to a POC test and several such tests have recently been evaluated as part of large multi-site consortiums in adults and children. Recent work by us and others have shown the potential of host mRNA transcripts for diagnosis of TB using the Cepheid GeneXpert platform (4–6) and host protein markers have also been incorporated into lateral flow tests for detection of diseases such as leprosy, TB and COVID-19 (7, 8). Lateral flow tests are cheap ($1-2/test) and do not require expertise, making them ideal for use in resource-limited settings. A recent study has shown a combination of CAD with the GeneXpert host-response test improved overall accuracy for TB with an increase in specificity from 70 to 79% compared to CAD alone, when sensitivity was set at 90% for a triage test (3). This suggests the need for optimised algorithms in addition to optimised biomarker signatures. We have previously shown the potential for sputum-derived host biomarkers to diagnose TB due to higher levels of *ex vivo* cytokines compared to serum. Combination of FGF, IL-13 and IFN-γ resulted in 96% correct classification of TB (sensitivity 85% and specificity 96%) (9). However, the sample size was limited, and no training and test set division could be performed. There were also too few smear-negative subjects to determine accuracy of the signature in this difficult to diagnose group. The aim of this study was therefore to determine the accuracy of this signature in a larger sample size, identify optimal signatures for diagnosis and treatment response, and determine applicability for use in children, and in samples from non-African countries.

## Materials and methods

### Study participants

Study participants included adults and children recruited from the adult or childhood TB clinic at MRCG with symptoms suggestive of TB but prior to clinical or microbiological confirmation. Inclusion criteria included cough of minimally 2 weeks for adults or any duration for children plus one other symptom suggestive of TB including fever, night sweats or unexplained weight loss. Sputum samples were collected at recruitment from all participants and at 1 month and 2 months from those who were subsequently found to have TB. Microbiological tests (smear microscopy and/or culture and/or GeneXpert) were performed at baseline and follow up for those on TB treatment. Patients who were negative were classified as having other respiratory diseases (ORD), which consisted approximately 50% of acute respiratory infections and 50% of chronic lung diseases, including asthma and COPD. Participants who were smear negative without any further confirmatory tests available (n=200) had a mycobacterial load assay (MBLA) performed to confirm the smear results (10). These participants were classified as probable ORD since MBLA is not a WHO-approved diagnostic assay but were combined with confirmed ORD (those with at least 2 negative microbiological tests) for analysis purposes. In addition, samples from the FIND biobank were obtained from 3 other countries (South Africa, Peru and Vietnam) in order to compare results with our Gambian cohort. These samples were defined as definite TB (confirmed culture positive), or other respiratory disorders (ORD; confirmed culture negative), and cryopreserved sputum pellets were shipped to MRCG laboratories for downstream processing. For analysis of treatment response, fast responders were defined as patients converting to culture negative by 2 months and slow responders were defined as converting by 6 months of therapy.

### Molecular bacterial load assay

Levels of ^16^S RNA and internal control (IC) were quantified using reverse transcription polymerase chain reaction (RT-PCR). To detect ^16^S RNA, a master mix containing 12.5 μl Quantitect Master Mix, 6.65 μl of nuclease free water, 0.25 μl reverse transcriptase, 0.3 μl of ^16^S-ROX (Rox-AGGACCACGGGATGCATGTCTTGT-BHQ2) (all supplied by Qiagen, Netherlands) per reaction was prepared. A master mix containing 12.5 μl Quantitect Master Mix, 5.05 μl of nuclease free water, 0.25 μl reverse transcriptase, 1.2 μl 50nM MgCl^2+^(Qiagen, The Netherlands) and 1 μl VIC labelled 560 Control Mix (Bioline, UK) per reaction was prepared to detect the IC. 5μl of RNA standards, samples or H_2_0 were added to the 96 well plate in triplicates; 20 μl of ^16^S-ROX mastermix was added to two wells, 20 μl of IC mastermix was added to the third. The plate was briefly vortexed, then centrifuged at 10,000 rpm for 30s before being placed in 7500 Real-Time PCR System (Applied Biosystems, USA). The cycling parameters were set to 50°C for 30 mins, 95 °C for 10 mins, then 45 cycles of 95°C for 15 secs and 60 °C for 1 min. Analysis was performed on ABI 7500 software (version 2.3, Affymetrix, USA).

### Sputum digestion

Sputum digestion was performed on fresh samples (Gambian analysis only) or on thawed cryopreserved native sputum samples (Gambia, Peru, South Africa, and Vietnam for analysis of global signatures). 1 ml of sputum was diluted with an equal volume of a 1:10 stock dilution of Sputolysin^®^ (Calbiochem, USA). The solution was mixed vigorously for 30 seconds and incubated at room temperature for 15 minutes. Following incubation, digested sputum was centrifuged (2000rpm for 10 minutes), and supernatants were harvested and stored at -80°C until use.

### Multiplex immunoassays

Two multiplex immunoassays were performed for each sample: 1) a 27-plex Bio-Plex Pro™ Human T helper (Th)1/Th2 Cytokine Panel from Bio-Rad (Belgium) with analytes: Interleukin (IL)-1β, IL-1rα, IL-2, IL-4, IL-5, IL-6, IL-7, IL-8, IL-9, IL-10, IL12p70, IL-13, IL-15, IL-17A, Eotaxin, Fibroblast Growth Factor (FGF)-basic, Granulocyte-colony stimulating factor (G-CSF), Granulocyte-macrophage (GM)-CSF, Interferon (IFN)-γ, IFN-y induced protein 10kDa (IP-10), Monocyte Chemoattractant Protein (MCP)-1, Macrophage inflammatory protein (MIP)-1α, MIP-1β, Platelet derived Growth Factor (PDGF)-BB, Regulated on activation, normal T-cell expressed and secreted (RANTES), Tumour necrosis factor (TNF)-α and Vascular Endothelial Growth Factor (VEGF) and 2) a Bio-Plex ProTM Human inflammation panel I/II 37-plex kit (Bio-Rad, Belgium) with analytes: Tumour necrosis factor super family (TNFSF)13, TNFSF13B, TNFRSF8, soluble cluster of differentiation (sCD)163, Chitinase 3-like 1, soluble IL-6 receptor (slL-6R)β, IFN-α2, IFN-β, IFN-y, IL-2, slL6Rα, IL-8, IL-10, IL-11, IL-12(p40), IL-12(p70), IL-19, IL-20, IL-22, IL-26, IL-27(p28), IL-28A/IFN-λ2, IL-29/IFN-λ1, IL-32, IL-34, IL-35, TNFSF14, matrix metalloproteinase (MMP)-1, MMP-2, MMP-3, Osteocalcin, Osteopontin (OPN), Pentraxin-3 (PTX), sTNF-R1, sTNF-R2, Thymic Stromal Lymphopoietin (TSLP) and TNFSF12. Analysis was performed according to the manufacturer instructions.

### Statistical analysis

The AUC, log2 fold change and Wilcoxon test p-values were calculated for differences in each cytokine between TB and ORD groups. P-values were adjusted for multiple testing with the Benjamini-Hochberg method (11). Generalised linear multiple regression analyses were performed, and receiver operator characteristics curves (ROC) generated to assess the predictability and performance of the analytes in differentiating TB from ORDs. The performance characteristics and cut-offs for individual markers of the ROC analyses were generated using OptimalCutpoints-package version 1 in association with the Youden Index method. For signature identification, a training set was created with 2/3 of the samples from each group and a test set with the remaining samples. A random forest model was generated based on all cytokines in the training set with results presented from the test set only. For validation of our previous signature, only 3 markers were included in the model. For analysis of treatment response, samples from confirmed TB patients were analysed using longitudinal Wilcoxon Ranked sum test and Friedman test.

## Results

### Participant demographics

For samples from Gambia, a total of 741 participants were included. Of these, 428 were confirmed to have TB, while 313 had probable ORD. Of the confirmed TB cases, 30% were female compared to 48% for the ORD group. The median age (interquartile range) was 31 (23-43) years and 40 (24-56) years for TB and ORD groups respectively (p<0.0001) (**Supplementary Table 1**). For the global analysis, samples from 414 HIV negative adults were received from the FIND biobank (221 TB, 414 ORD). There was no significant difference in the % of females per group but no information on the age range was available (**Supplementary Table 2**).

### Univariate analysis of sputum host markers

**Figure 1:**
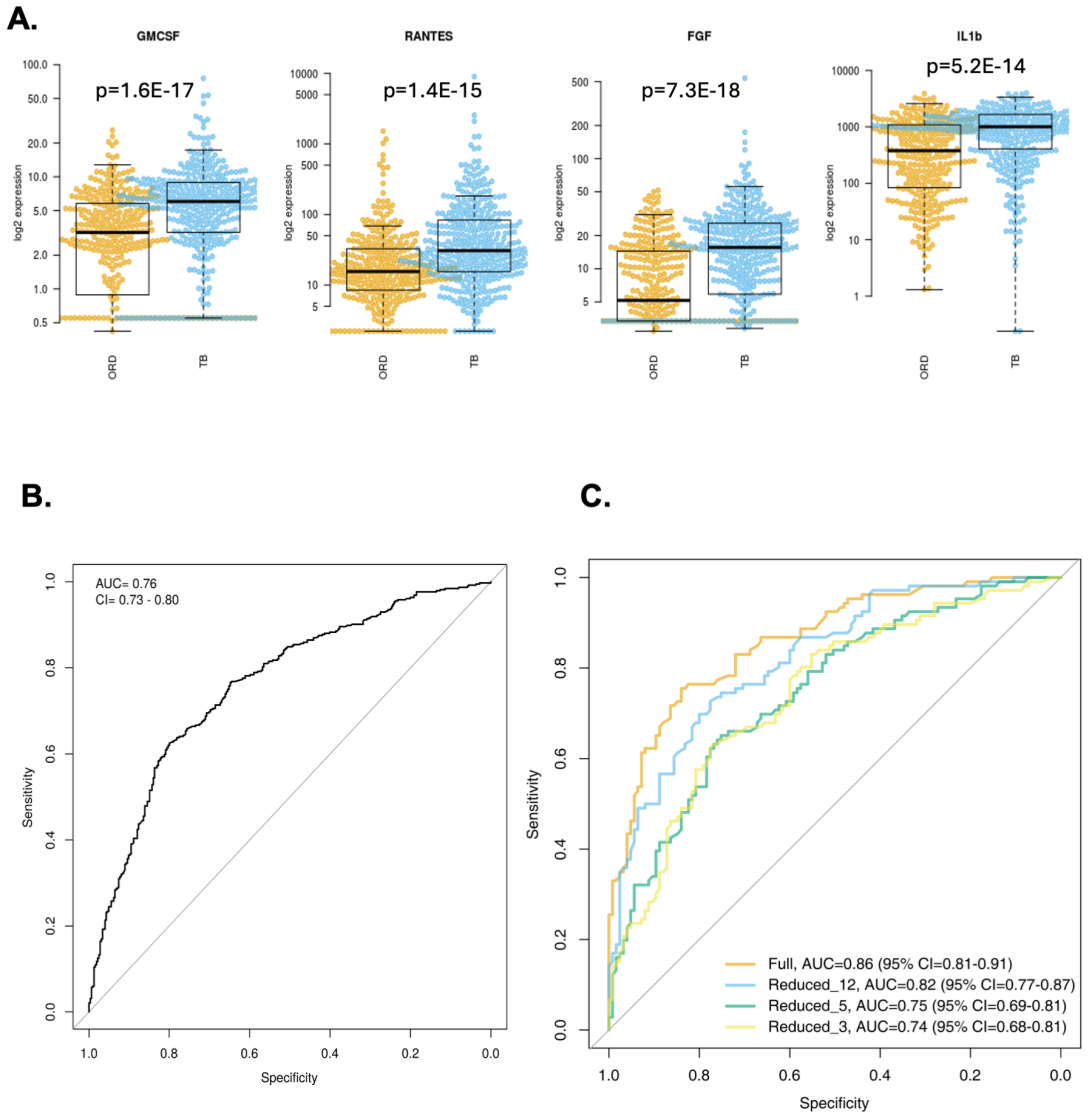
Analysis of Gambian samples. **(A)** Top 4 markers for discriminating TB from ORD in Gambian samples (from left, GMCSF, RANTES, FGF, IL-1β). Data were analysed using Wilcoxon test and final concentrations were adjusted using Log2 expression (pg/ml). P-values are indicated. Line = median. Orange = ORD, blue = TB. **(B)** ROC curve of original 3 marker signature (FGF, IL-13 and IFN-γ). **(C)** ROC curves of newly derived signatures: Full set (AUC 0.86; orange line), reduced 12-marker signature (AUC 0.82; blue line), reduced 5-marker signature (AUC 0.75, green line) and reduced 3-marker signature (AUC 0.74, yellow line).

For Gambian participants, 37 analytes were significantly different between TB and ORD patients irrespective of their HIV status (**Table 1**) with most markers showing higher levels in TB patients. Only APRIL showed a significantly higher level in ORD compared to TB with a concentration of 1103pg/ml and 118268pg/ml for TB and ORD respectively (p=7.24E-08; **Table 1**). The 4 best analytes were GMCSF, RANTES, FGF and IL-1β (**Figure 1A**). When ROC analysis was performed, MMP-2 was the overall best performer with an AUC of 0.73 (sensitivity 58% and specificity 81%) with a high concentration cut-off of 9273 pg/ml (**Table 1**). When the original 3-marker signature was analysed (FGF, IL-13 and IFN-γ), performance was poorer than in the original analyses, with an AUC of 0.76 (CI = 0.73 - 0.80) (**Figure 1B**).

**Table 1:** Univariate analysis.

Marker	TB	ORD	Adjusted p-value	Sens (%)	Spec (%)	AUC
IL-9	200	159	2.47E-04	85	32	0.58
IL-8	18067	12049	3.77E-05	62	55	0.59
IL-15	325	210	2.55E-05	72	49	0.59
IL-13	13	7	9.71E-06	75	45	0.60
IL-6	34	14	3.39E-06	67	54	0.61
MCP-1	144	60	1.39E-07	72	48	0.61
PDGF	158	108	1.26E-07	36	80	0.61
Eotaxin	30	13	7.16E-08	67	54	0.63
IFN-γ	279	123	3.90E-09	66	59	0.64
IL-4	11	5	7.86E-11	64	61	0.65
IL-5	20	10	1.42E-11	65	64	0.65
TNF-α	672	106	7.44E-11	76	51	0.65
MIP-1α	9	3	1.14E-13	78	49	0.67
IL-2	26	7	5.82E-13	73	56	0.67
IL-17A	95	22	1.62E-13	71	58	0.67
RANTES	30	15	1.49E-15	68	64	0.68
IL-1β	997	310	1.49E-15	71	61	0.69
FGF	15	5	1.49E-15	61	70	0.69
GMCSF	6	3	1.49E-15	60	73	0.69
MMP-3	535	442	4.19E-02	82	28	0.55
sIL-6Rα	133	70	2.17E-03	38	77	0.58
IL-8	18200	5299	3.12E-05	78	42	0.60
APRIL	1103	118268	7.24E-08	67	60	0.63
sTNFR1	120	22	1.90E-09	57	68	0.64
sTNFR2	122	33	4.16E-10	64	62	0.65
BAFF	9753	2612	4.07E-15	82	50	0.68
MMP-2	13683	2266	4.07E-15	56	80	0.73
TNF-α	40	10	5.73E-03	46	71	0.60
MCP-1	490	120	5.73E-03	57	60	0.61
MIP-1β	80	59	8.94E-05	42	36	0.64
MIP-1α	74	3	3.03E-05	53	70	0.65
IL-1β	851	218	3.03E-05	59	69	0.66

TB, tuberculosis; ORD, other respiratory diseases; AUC, Area under the receive operator curve; Sens, sensitivity; spec, specificity.

### Identification of signatures for TB triage in Gambian adults and children

For identification of adult pulmonary TB, regardless of HIV status, we identified an 8-marker signature consisting of FGF, IL-1β, IL-8, IL-10, IL-12p70, IL-13, MIP-1β and VEGF which gave an AUC of 0.78 (95% CI: 0.75, 0.84), sensitivity of 79% (95% CI: 75, 84) and specificity of 67% (95% CI: 60, 73) (**Table 2**). Importantly, a 3-marker signature (IL-1β, IL-7 and VEGF) classified smear negative but culture positive TB from ORD with an AUC of 0.74, sensitivity of 86% and specificity of 60%. When only HIV uninfected individuals who were both smear and culture positive were analysed, a different 8-marker signature consisting of IFN-y, IL-1β, IL-8, IL-10, IL-12p70, MIP-1β, RANTES and VEGF was identified which resulted in an AUC of 0.77. When participants <18 were analysed, a four-marker signature consisting of FGF, IL-4, MIP-1α and RANTES was identified which gave an AUC of 0.87(0.95% CI: 0.75, 0.99), sensitivity of 82% (95% CI: 64,100) and specificity of 87% (95% CI: 73, 100) (**Table 2**).

**Table 2:** Biosignature classification.

Biosignature	AUC (95% CI)	Sens (95% CI)	Spec (95% CI)
Only adults			
FGF, IL-1β, IL8, IL10, IL-12p70, IL-13, MIP-1β and VEGF	0.78 (0.75, 0.84)	79 (75, 84)	67 (60, 73)
	0.67 (0.57, 0.77)	67 (56, 78)	67 (54, 80)
All patients			
Eotaxin, FGF, GMCSF, IFN-y, IL-1β, IL-8, IL-10, IL-12p70, IL-13, PDGF, RANTES, VEGF	0.81 (0.77, 0.84)	75 (71, 80)	76 (70, 81)
	0.71 (0.63, 0.80)	55 (44, 66)	90 (82, 97)
Adult smear and culture positive excluding HIV infected			
IFN-y, IL-1β, IL-8, IL-10, IL-12p70, MIP-1β, RANTES, VEGF	0.77 (0.72,0.82)	78 (73, 84)	70 (64, 77)
	0.77 (0.68,0.87)	78 (66, 89)	69 (57, 82)
Smear negative – culture positive only			
IL-1β, IL-7, VEGF	0.74 (0.60, 0.87)	86 (71, 100)	60 (45, 75)
HIV infected only			
VEGF, RANTES	0.78 (0.65, 0.91)	56 (32, 81)	90 (80, 99)
Children			
FGF, IL-4, MIP-1α, RANTES	0.87 (0.75, 0.99)	82 (64,10)	87 (73, 100)

AUC, area under the receive operator curve; Sens, sensitivity; spec, specificity; CI, confidence interval.

We next divided our full dataset (combined adults and children) into a training and test set. A training set was created with 2/3 of the samples from each group and a test set with the remaining samples. A random forest model was generated based on all cytokines in the training set resulting in an AUC of 0.86, sensitivity of 76% (66-83) and specificity of 84% (76-90). The number of variables necessary to achieve full power was 12 analytes (MMP-2, TNFSF13B, RANTES, GMCSF, FGF, MIP-1β, MMP-1, MIP-1α, IL-1β, IL-2, TNF-α, Chitinase3like1) with an AUC of 0.82, sensitivity of 73% (63-81) and specificity of 78% (69-85). A further reduced model with only 3 analytes (MMP = 2, TNFSF13β and RANTES) resulted in an AUC of 0.74, sensitivity of 73% (63-81) and specificity of 78% (69-85) (**Figure 1C**).

### Identification of a global cytokine signature

**Figure 2:**
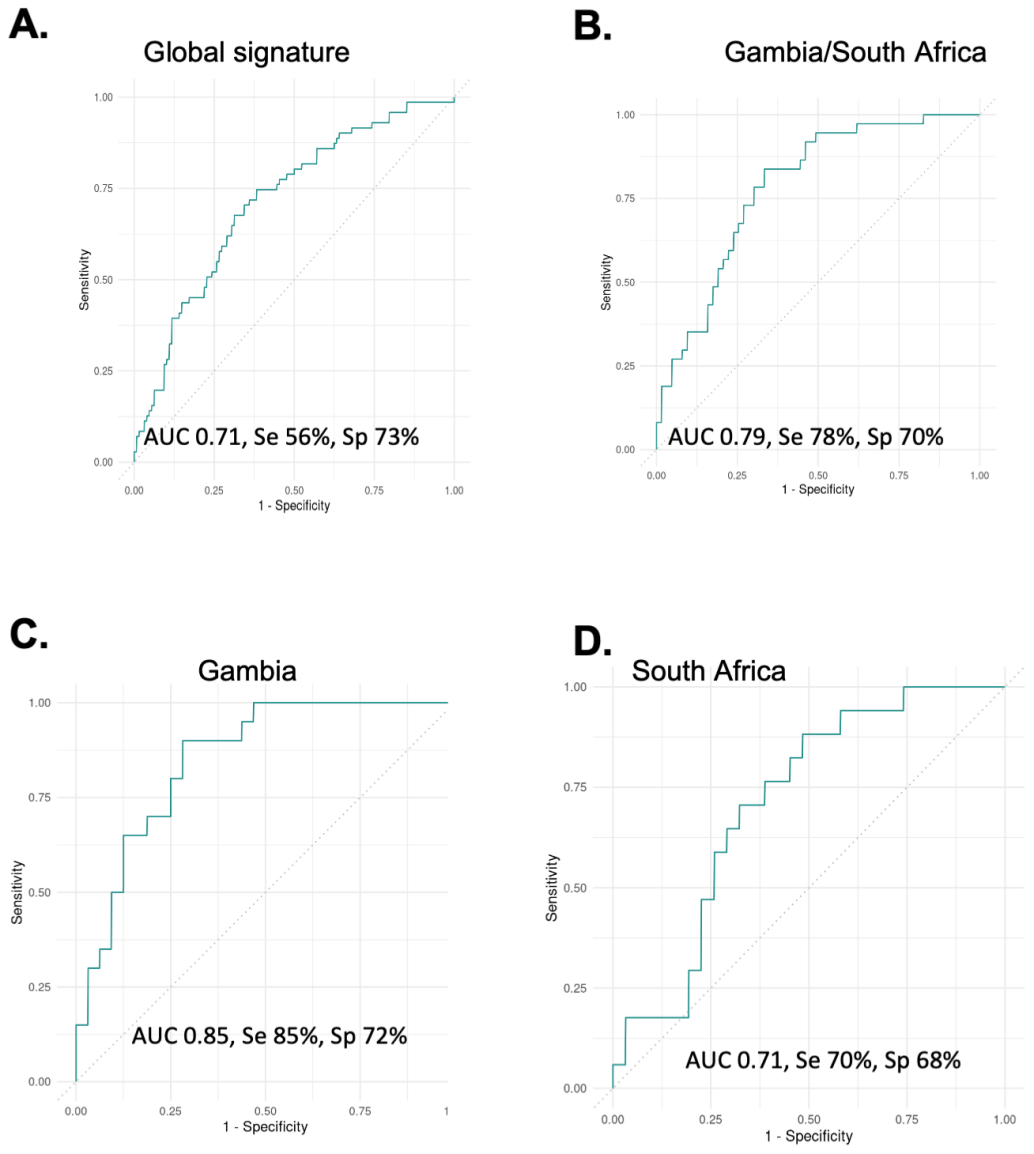
Analysis of global samples. **(A)** all samples from Vietnam, Peru, South Africa and Gambia combined showing performance for the best signature (5-markers) with an AUC of 0.71. **(B)** Gambia and South African samples showing performance of a different 5-marker signature with an AUC of 0.79. **(C)** The same signature on Gambia samples alone with an AUC of 0.85 and **(D)** the same signature on South African samples alone with an AUC of 0.71.

We next analysed adult TB and ORD samples from the FIND biobank. Levels of TNF-α (p<0.0001), MMP-2 (p<0.0001), IL-1β (p=0.0002), IL-22 (p=0.0004) and LIGHT/TNFSF14 (p=0.0007) were all significantly higher in sputum from TB compared to ORD patients when all 4 countries were combined (not shown). A ‘global’ signature was identified after dividing our data into training and test sets. This 5-marker signature consisting of FGF, OPN, MMP-1, TNF and IL-1β resulted in an AUC of 0.71, sensitivity of 56% and specificity of 73% (**Figure 2A**). When only Gambian and South African samples were analysed (pan-African), a 5-marker signature consisting of IL-11, IL-1β, IL-2, IL-6 and TNF-α resulted in an AUC of 0.79, sensitivity of 78% and specificity of 70% (**Figure 2B**). This same signature in samples from Gambia alone resulted in an AUC of 0.85, sensitivity of 85% and specificity of 72% (**Figure 2C**), with worsening performance seen in samples from South Africa alone (AUC 0.71; **Figure 2D**).

### Treatment response

To assess the potential usefulness of the markers to monitor treatment response, Wilcoxon Ranked sum test and Friedman test were used to assess the changes in level during treatment. Of adult patients confirmed to have TB and who returned for follow-up visits, 39 provided good quality sputum at month 2. Of these, 15 (3.5%) remained culture positive while the rest were microbiologically negative. At 2 months,13 markers were significantly lower compared to baseline (EGF, Eotaxin, TGF-α, GM-CSF, IL-1α, IL-1β, IL-8, MDC, MIP-1α, MIP-1β, TNF-β, and MCP-1) (not shown). When fast versus slow responders were analysed, 14 markers (EGF, FGF-2, TGF-α, GMCSF, Fractalkine, IFN-y, IL-10, MCP-3, IL-12p40, IL-12p70, IL-15, sCD40L, IL-2, IL-7, MCP-1, TNF-α and TNF-β) were observed to discriminate people who responded faster to treatment from the slow responders (not shown). We identified a four-marker signature consisting of EGF, IL-15, MIP-1β and TNF-β that could predict slow versus fast treatment responders at baseline with an AUC of 0.74, sensitivity of 75% and specificity of 80% (**Table 3**).

**Table 3:** Fast versus slow treatment response biosignature.

Biosignature	AUC (95% CI)	Sensitivity (95% CI)	Specificity (95% CI)
EGF, IL-15, MIP-1β, TNF-β	0.74 (0.57, 0.91)	75 (54, 96)	80 (75, 86)

AUC, area under the receiver operator curve; CI, confidence interval.

## Discussion

The availability of a cheap and rapid TB triage test is essential for better patient management, reduction in morbidity and mortality, and blocking of the transmission chain. Here we show that host cytokines present in *ex vivo* sputum samples can be used to screen for TB amongst adults and children who present with symptoms of respiratory disease at local health clinics. We identified several signatures for diagnosis of TB in adults, children, and for monitoring treatment response. Importantly, our control group (ORD) consisted of patients presenting with respiratory symptoms, but confirmed not to have TB. Thus our signature is specific to TB and not just a general inflammatory response.

The best performing single analyte was MMP-2, resulting in an AUC of 0.73. However, this is not good enough for a screening test, indicating the requirement for a multi-analyte signature. We identified an 8-marker signature for diagnosis of HIV negative smear positive and culture positive TB with sensitivity of 78% and specificity of 69%. In addition, we identified a 3-marker signature (IL-1β, IL-7 and VEGF) which could diagnose smear negative but culture positive TB with an accuracy of 74% and 71% respectively. Importantly, we also identified a 4-marker signature (FGF, IL-4, MIP-1α and RANTES) that could discriminate children with confirmed TB from those with unlikely TB with an AUC of 0.87, a sensitivity of 82% and specificity of 87%. A previous, larger study from The Gambia identified a 3-marker signature following overnight incubation of blood resulted in an AUC of 0.74, a sensitivity of 72% and specificity of 75% (12). The majority of children in the present study were old enough to produce spontaneous sputum (median age 15 years), with only 4 children ≤5 years old, who required induced sputum. Importantly, however, the induced sputum samples still showed high levels of cytokines.

Interestingly, when we divided our data into a training and test set, we identified a 12-marker signature with an AUC of 0.82, while a reduced model with only 3 analytes (MMP-2, TNFSF13B and RANTES) resulted in an AUC of 0.74. Whilst these findings suggest potential for use as a triage test, neither signature reached the required WHO target product profile. This may be due to the relatively small sample size for this study, plus the inclusion of several sub-groups such as smear negative cases, people living with HIV, and children. Most of these sub-groups were too small to analyse on their own. However, the results hold promise and should be analysed in larger cohorts with enough power for each sub-analysis. Importantly we did analyse samples from multiple countries with results showing that a pan-African signature gave the best performance. This is interesting as our work with mRNA signatures has also shown the best performance in African countries, potentially due to differences in host and pathogen genetics, and country-specific diagnostic, health seeking behaviours and environmental factors. However, due to differences in sample collection and processing, it would be important to perform this analysis in fresh samples from multiple countries using a standardised approach.

We also identified a 4-marker biosignature consisting of EGF, IL-15, MIP-1β and TNF-β that could differentiate TB patients into slow or fast treatment responders at baseline in relation to smear or culture outcome at 2-months with a sensitivity of 75% and specificity of 80%. It is interesting to note that these markers are different to those identified for diagnosis of TB which mainly reflects the inflammatory response. Median levels of all markers were elevated in slow responders, suggesting an on-going immune response (with IL-15, MIP-1β and TNF-β) while elevated EGF indicates tissue repair and thus has implications in long-term lung pathology. The ability to classify patients’ treatment response will help to provide patient-centred treatment plans. Currently we are reliant on sputum culture negativity at 2 months, yet its predictive ability is limited, and culture also has significantly delayed turnaround time. It is also very difficult to obtain quality sputum during the course of treatment, therefore, the availability of markers at baseline that can predict the outcome of treatment would help to mitigate this problem. However, our data are limited by not knowing the outcome of the participants in terms of relapse/reinfection. Future work to determine if this 4-marker signature can also predict future relapse is needed before further development as a point-of-care test.

The main limitation of this study is the relatively small sample size, particularly for subgroup analysis (HIV+, children). In addition, follow up of the non-TB group should be performed to determine if any individuals became TB positive later. Whilst our results are promising, they require validation in larger multi-site studies with standardised protocols for sample collection and processing and with head-to-head comparison of blood and sputum markers. The majority of studies assessing potential host markers for a triage test have focussed on blood as this sample type is universal, compared to sputum which cannot be produced by everyone. However, we have previously shown that cytokine levels are significantly higher in sputum than serum, being a surrogate for the site of infection, thus making it more likely to translate to a lateral flow test. In addition, levels were high whether induced or spontaneous sputum. Thus, whilst non-sputum-based tests are ideal for a POC test, due to issues with sputum quality and potential infection risk, our results suggest that sputum tests based on analysis of the host rather than the pathogen, particularly with a signature that performs well in smear negative individuals, have potential for development of rapid POC testing at basic health clinics. Where CXR analysis is also available, AI imaging could also be combined to enhance performance. However, whilst the results are promising for regional-specific and treatment response signatures, our pilot data do not support development of a global test for TB triage based on sputum host signatures.

## Data Availability

The raw data supporting the conclusions of this article will be made available by the authors, upon request to the authors.
