# Supplementary material for: Sputum host cytokine signatures for diagnosis of TB in children and adults
Source: Front Immunol. 2025 Oct 14;16:1652719. doi: 10.3389/fimmu.2025.1652719 (PMC12558920; doi:10.3389/fimmu.2025.1652719)
Supplement: Supplementary file 1 [file Table1.docx]

**Supplementary Table 1: Gambian participant demographics**

| **Patient characteristics** | **TB** | **ORD** |
| --- | --- | --- |
| n | 428 | 313 |
| Female n (%) | 128 (30) | 149 (48) |
| HIV infected n (%) | 16 (4) | 4 (1) |
| Children (<18 years) | 17(4) | 23 (7) |
| Median IQR age (years) | 31(23-43) | 40 (24-56) |

**Supplementary Table 2: Global sample demographics**

|  | Vietnam | South Africa | Peru |
| --- | --- | --- | --- |
| ORD | 100 | 90 | 100 |
| TB | 50 | 50 | 50 |
| Female n (%) | 59 (61) | 68 (51) | 73 (51) |
| Age | Adult | Adult | Adult |
| HIV infected | 0 | 0 | 0 |
